# Biomarker Discovery of Pancreatic and Gastrointestinal Cancer by 2DICAL: 2-Dimensional Image-Converted Analysis of Liquid Chromatography and Mass Spectrometry

**DOI:** 10.1155/2012/897412

**Published:** 2012-07-10

**Authors:** Masaya Ono, Masahiro Kamita, Yusuke Murakoshi, Junichi Matsubara, Kazufumi Honda, Banno Miho, Tomohiro Sakuma, Tesshi Yamada

**Affiliations:** ^1^Division of Chemotherapy and Clinical Research, National Cancer Center Research Institute, 5-1-1 Tsukiji, Chuo-ku, Tokyo 104-0045, Japan; ^2^Third Department of Surgery, Tokyo Medical University, 6-7-1 Nishi-shinjuku, Shinjuku-ku, Tokyo 160-0023, Japan; ^3^Institute for Stem Cell Biology and Regenerative Medicine (ISCBRM) and Stanford Cancer Center, Stanford University, 265 Campus Drive, Room G2015, Stanford, CA 94305, USA; ^4^Bio Science Department, Research and Development Center, Mitsui Knowledge Industry Co., Ltd., 2-7-14 Higashinakano, Nakano-Ku, Tokyo 164-8555, Japan

## Abstract

Biomarkers tested by blood sample are of great use to clinicians as they provide useful information to aid an early and accurate diagnosis. Comprehensive “omics” studies are expected to facilitate the identification of such new biomarkers, and much research is being performed in this area. Our proteomics analysis system of 2-dimensional image-converted analysis of liquid chromatography and mass spectrometry (2DICAL) has successfully identified several new blood biomarkers from the clinical blood samples of pancreatic and colorectal cancer patients.

## 1. Introduction

Proteomic studies are powerful tools for identifying useful new biomarkers, and much research is currently being performed in this area. However, the blood proteome is extraordinary difficult to analyze because protein concentrations can vary by 12 orders of magnitude [1]. Thus, biomarker discovery using proteomics requires the development of effective pretreatment protocols to reduce the complexity of blood samples. The identification of biomarkers from clinical samples generally needs large numbers of samples to be compared. The same is true for the identification of biomarkers by mass-spectrometry-coupled proteomics [2, 3]. Our proteomics analysis system of 2-dimensional image-converted analysis of liquid chromatography and mass spectrometry (LC/MS; 2DICAL) and the procedure for reducing blood sample complexity have overcome these problems. We report the successful discovery of several new blood biomarkers for pancreatic and colorectal cancer [4, 5].

## 2. Biomarker Detection

### 2.1. Recruitment of Clinical Samples

For biomarker discovery, it is important to collect quality-controlled blood samples. We developed a multi-institutional protocol to preserve blood condition during sampling, storing, freezing, and thawing; all samples were collected and managed at the National Cancer Center Research Institute [6].

### 2.2. Sample Preparation

As the concentrations of different blood proteins can vary by over 12-orders of magnitude, it is essential to remove abundant proteins or to concentrate specific proteins before proteomics analysis. For this purpose, we used lectin affinity column [5, 7], major protein removal column [8], and hollow fiber membrane (HFM) [9, 10].

### 2.3. Biomarker Discovery

To identify candidate biomarkers from the proteomics data, we utilized our 2DICAL analysis system that performs a quantitative comparison of unlabeled shotgun proteomics data generated by LC/MS and enables biomarker discovery from a large number of clinical samples. For selecting blood biomarkers, several decades of samples from cancer patients and healthy controls were analyzed by 2DICAL.

### 2.4. Biomarker Verification

Biomarkers selected by 2DICAL must be verified. As a rule, we first confirmed 2DICAL results using specific antibodies in small-scale immunoblotting assays. Once marker expression was detected and differences in the expression between patient and control samples were confirmed, large-scale verification was conducted. For this purpose, we used in-house reverse phase protein microarrays (RPPA), which can simultaneously assess hundreds of blood samples by antibody staining [11, 12]. Validation at the hundred-sample scale by multiple reaction monitoring/selective reaction monitoring (MRM/SRM) [13] is also ongoing.

## 3. Novel Applications of Analysis

We developed our original application for biomarker identification, that is, 2DICAL and RPPA.

### 3.1. 2DICAL

2DICAL was developed as a shotgun proteomics analysis system. It analyzes the data of mass to charge ratio (*m/z*), peak intensity, retention time (RT), and each sample generated by LC/MS as the elemental data; it deploys various 2-dimensional images with different combinations of axes using these four elements. From the *m/z*-RT image, peaks derived from the same peptide in the direction of acquiring time are integrated. By adding algorisms to ensure reproducibility of *m/z* and RT, the same peak can be compared precisely across different samples, and a statistical comparison of identical peaks in different samples leads to the discovery of specific differentially expressed peptide peaks. Specific peaks are designated by their *m/z* and RT coordinates, and further analysis is based on these identifiers. Isotopic labeling is not necessary, and large numbers of samples can be analyzed in this way [4, 8].

### 3.2. RPPA

RPPA is an emerging high-throughput proteomics technique for validating new biomarkers [14, 15]. Furthermore, RPPA requires significantly lower amounts of clinical samples for quantification than established clinical tests such as enzyme-linked immunosorbent assay (ELISA). We made in-house RPPA using ProteoChip glass slides (Proteogen, Seoul, Republic of Korea) to test hundreds of blood samples simultaneously. For this technique, serially diluted samples are randomly plotted in quadruplicate in a 6,144-spot/slide format using a robot. The spotted slides are incubated with the primary antibody and biotinylated secondary antibody and then processed with a streptavidin-horseradish peroxidase conjugate. The stained slides are scanned on a microarray scanner. Statistical evaluation of the fluorescence intensity of individual samples is performed for large-scale validation of biomarker candidates [11, 12].

## 4. Biomarkers for Pancreatic Cancer and Gastrointestinal Cancer

Several blood biomarkers have already been discovered. Sample recruitment, sample preparation, biomarker discovery, and validation have been described for each biomarker.

### 4.1. Biomarkers for Pancreatic Cancer

Prolyl-hydroxylated *α*-fibrinogen [5] and CXC chemokine ligand 7 (CXCL-7) [10] were identified as pancreatic cancer biomarkers.

#### 4.1.1. Prolyl-Hydroxylated *α*-Fibrinogen

**Objective**

Screening for pancreatic cancer.

**Samples**

In total, 86 plasma samples (collected from 43 patients with pancreatic ductal adenocarcinomas and 43 healthy controls) were used for biomarker identification, and 273 plasma samples (collected from 160 patients with pancreatic ductal adenocarcinomas and 113 healthy controls) were used for validation.

**Sample Preparation**

Samples were treated with concanavalin A (Con A) to reduce plasma protein complexity.

**Biomarker Discovery**

**Figure 1:**
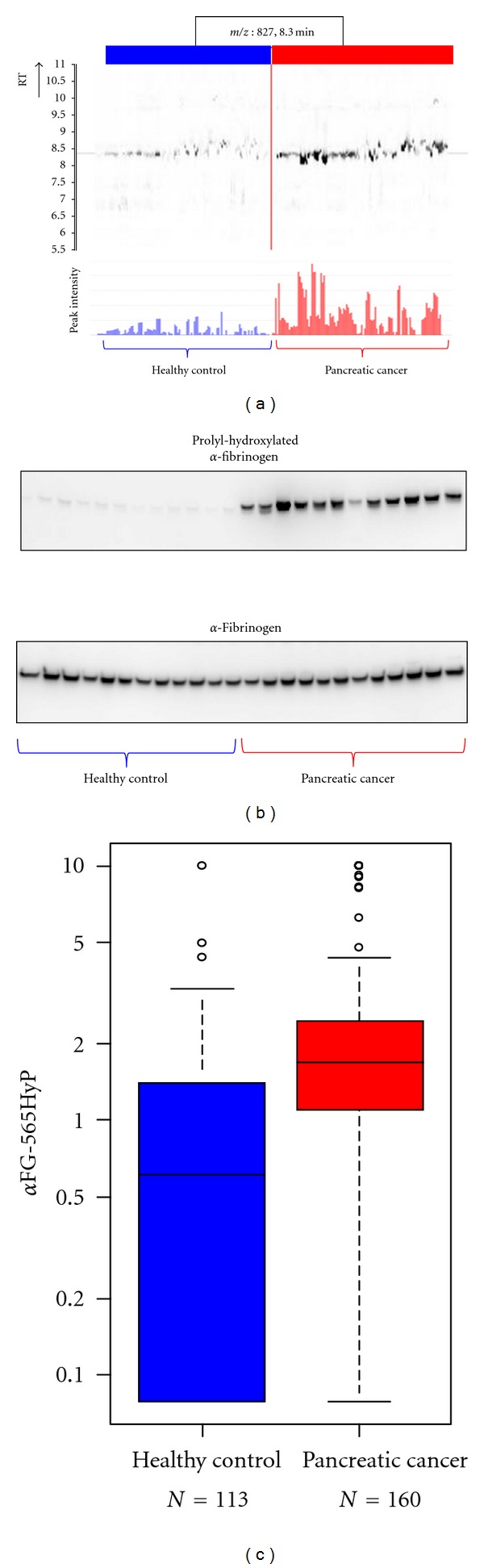
Discovery and validation of prolyl-hydroxylated *α*-fibrinogen as a pancreatic cancer biomarker (partially changed from [5]). (a) 2DICAL images of the peak (*m*/*z*, 827; RT, 8.3 min) with coordinates RT versus patients (upper) and intensity versus patients (lower). Red indicates samples from pancreatic cancer patients, and blue indicates samples from healthy controls. (b) Western blot of prolyl-hydroxylated *α*-fibrinogen (upper panel) and total *α*-fibrinogen (lower panel). (c) Large-scale ELISA validation of the plasma level of prolyl-hydroxylated *α*-fibrinogen using hundreds of clinical samples.

Samples were subjected to LC/MS and analyzed by 2DICAL. A total of 115325 peaks were detected, and 6 peaks of 412 *m*/*z* (RT 13.7 min), 546 *m*/*z* (8.3 min), 552 *m*/*z* (8.3 min), 827 *m*/*z* (8.3 min), 1141 *m/z* (29.0 min), and 1185 *m*/*z* (9.2 min) were statistically significant with >2 fold difference and *P* < 0.0005 (Mann-Whitney *U* test) between the pancreatic cancer patient group and healthy control group. Three of the 6 peaks were identified as hydroxyproline-modified *α*-fibrinogen fragments (Figure 1(a)).

**Biomarker Validation**

An antibody recognizing *α*-fibrinogen fragments with an ESSSHH P*GIAEFPSR (P*, 4-hydroxyproline) modification was generated and used for small-scale confirmation of the expression of prolyl-hydroxylated *α*-fibrinogen and the differences in the expression of modified protein between samples of pancreatic cancer patients and healthy controls (Figure 1(b)). A competitive ELISA was developed using this antibody to quantify plasma levels of prolyl-hydroxylated *α*-fibrinogen. A significant difference in prolyl-hydroxylated *α*-fibrinogen expression between plasma samples from pancreatic cancer patients and healthy controls was observed (*P* = 3.80 × 10^−15^, Mann-Whitney *U* test; Figure 1(c)).

#### 4.1.2. CXCL-7

**Objective**

Screening for pancreatic cancer.

**Samples**

A total of 45 plasma samples (collected from 24 patients with pancreatic ductal adenocarcinomas and 21 healthy controls) were used for biomarker discovery and 227 plasma samples (collected from 140 patients with pancreatic ductal adenocarcinomas and 87 healthy controls) were used for biomarker validation.

**Sample Preparation**

Samples were treated with HFM to reduce plasma protein complexity.

**Biomarker Discovery**

**Figure 2:**
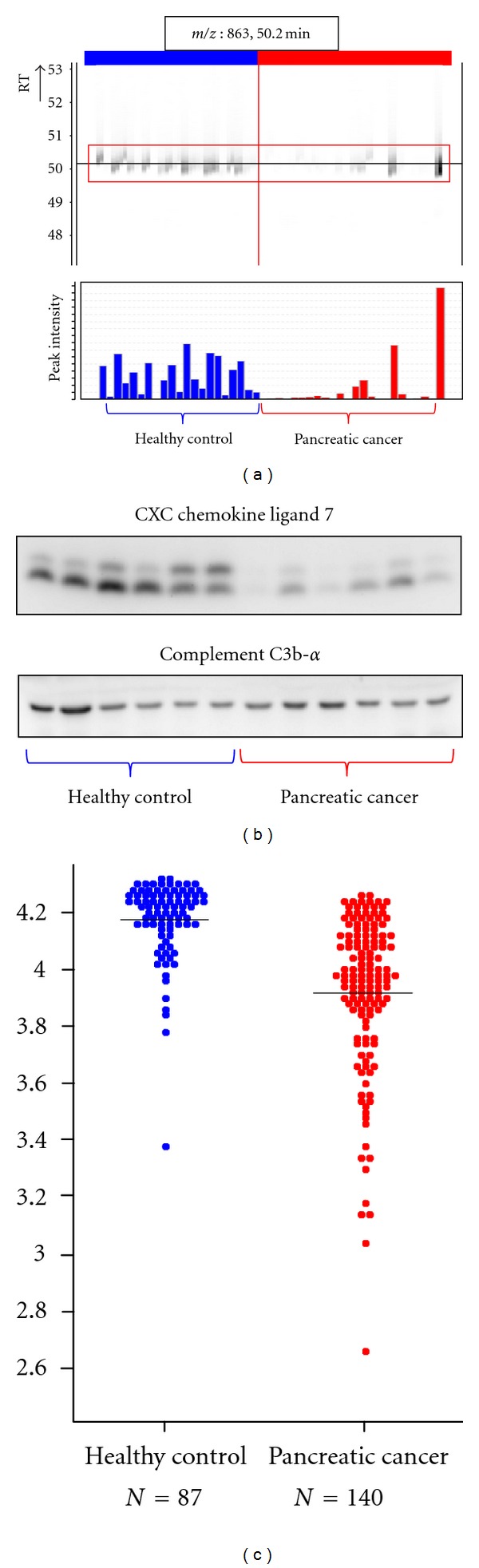
Discovery and validation of CXC chemokine ligand 7 as a pancreatic cancer biomarker (partially changed from [10]). (a) 2DICAL images of peptide peak (*m*/*z*, 863; RT, 50.2 min) with coordinates RT versus patients (upper) and intensity versus patients (bottom). Red indicates samples from pancreatic cancer patients, and blue indicates samples from healthy controls. (b) Western blot of CXC chemokine ligand 7 (upper panel) and the loading control Complement C3b-*α*. (c) Large-scale RPPA validation of the plasma level of CXC chemokine ligand 7 using hundreds of clinical samples.

Samples were subjected to LC/MS and analyzed by 2DICAL. A total of 53009 peaks were detected, and 140 peaks were differentially expressed between pancreatic cancer patients and healthy controls, with an area under curve (AUC) of >0.800. Of these, 10 proteins were annotated by database search of tandem mass spectra. The 862 *m*/*z* (RT 50.2 min) peak annotated as a fragment of CXCL-7 was specifically expressed in pancreatic cancer patients, with an AUC of 0.839 (*P* = 4.54 × 10^−5^ by Mann-Whitney *U *test) (Figure 2(a)).

**Biomarker Validation**

Small-scale confirmation of CXCL7 identification and differential expression was done by immunoblotting using an anti-CXCL-7 antibody (Figure 2(b)). For large-scale validation, 227 plasma samples were randomly plotted onto ProteoChip glass slides for RPPA and blotted with an anti-CXCL-7 antibody. CXCL7 expression in pancreatic cancer patients and healthy controls was confirmed to be significantly different (*P *= 1.40 × 10^−16^, Welch *t-*test; Figure 2(c)).

### 4.2. Biomarkers for Colorectal Cancer

Complement Component 9 (C9) [12] and adipophilin [16] were identified as colorectal cancer biomarkers.

#### 4.2.1. C9

**Objective**

Screening for colorectal cancer.

**Samples**

In total, 90 plasma samples (collected from 31 colorectal cancer patients and 59 healthy controls) were used for biomarker discovery, and 345 plasma samples (collected from 115 colorectal cancer patients and 230 healthy controls) were used for validation.

**Sample Preparation**

Samples were treated with a 12-abundant-plasma-protein removal columns to reduce plasma protein complexity.

**Biomarker Discovery**

**Figure 3:**
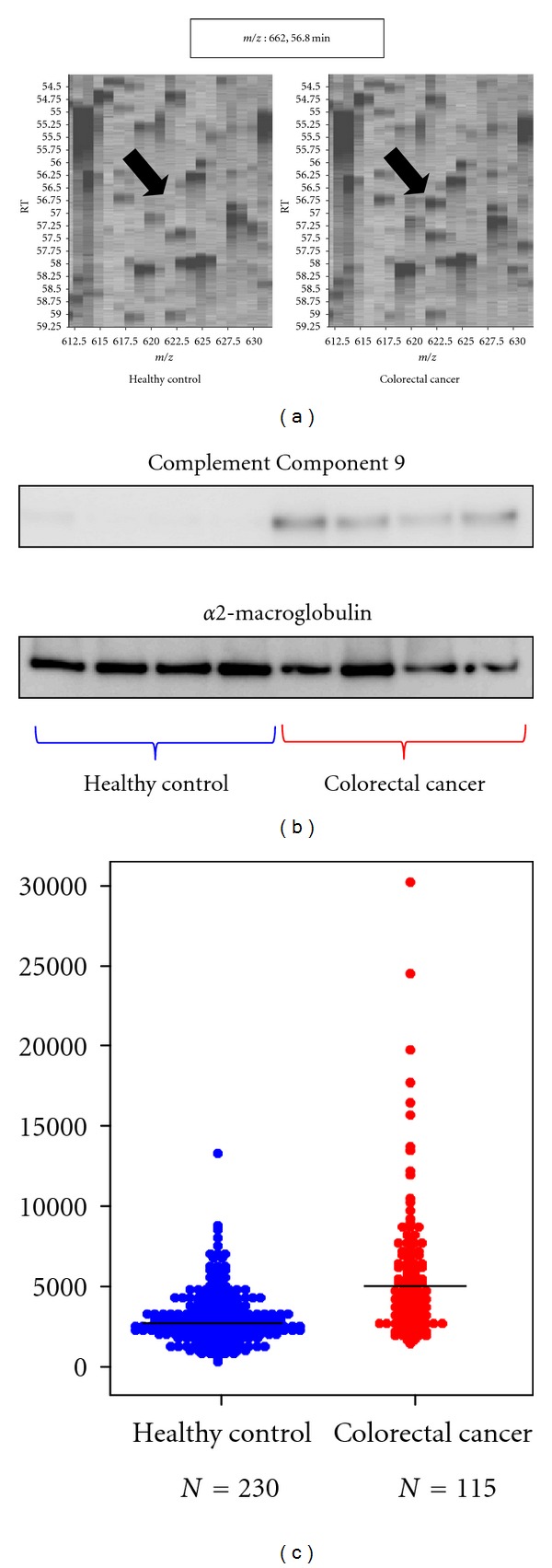
Discovery and validation of Complement Component 9 as a colorectal cancer biomarker (partially changed from [12]). (a) 2DICAL images with coordinates *m/z* versus RT. The intensity of the peak of 622 *m/z* and RT of 56.8 min (indicated by arrows) are clearly different in the plasma samples from healthy controls (left) and colorectal cancer patients (right). (b) Western blot of Complement Component 9 and the loading control *α*2-macroglobulin. (c) Large-scale RPPA validation of the plasma level of Complement Component 9 using hundreds of clinical samples.

Samples were subjected to LC/MS and analyzed by 2DICAL. A total of 94803 peaks were detected, and 90 peaks showed statistically significant differences in expression between plasma from colorectal cancer patients and healthy controls. Of these, 10 proteins were annotated by database search of tandem mass spectra. A peptide peak with 622 *m*/*z* (RT 56.8 min) was annotated as a fragment of C9 specific to colorectal cancer patients (*P* = 3.0 × 10^−5^, paired *t*-test; Figure 3(a)).

**Biomarker Validation**

Small-scale confirmation of C9 identification and differential expression was done by immunoblotting using an anti-C9 antibody (Figure 3(b)). For large-scale validation, 345 plasma samples were randomly plotted into ProteoChip glass slides for RPPA and blotted with an anti-C9 antibody. There was a significant difference in C9 expression in plasma from colorectal cancer patients and from healthy controls (*P* = 1.43 × 10^−12^, Student's *t*-test; Figure 3(c)).

#### 4.2.2. Adipophilin

**Objective**

Screening for colorectal cancer.

**Samples**

A total of 43 plasma samples (collected from 22 colorectal cancer patients and 21 healthy controls) were used for biomarker discovery, and 323 plasma samples (collected from 127 colorectal cancer patients and 196 healthy controls) were used for validation.

**Sample Preparation**

Samples were treated with HFM to reduce plasma protein complexity.

**Biomarker Discovery**

**Figure 4:**
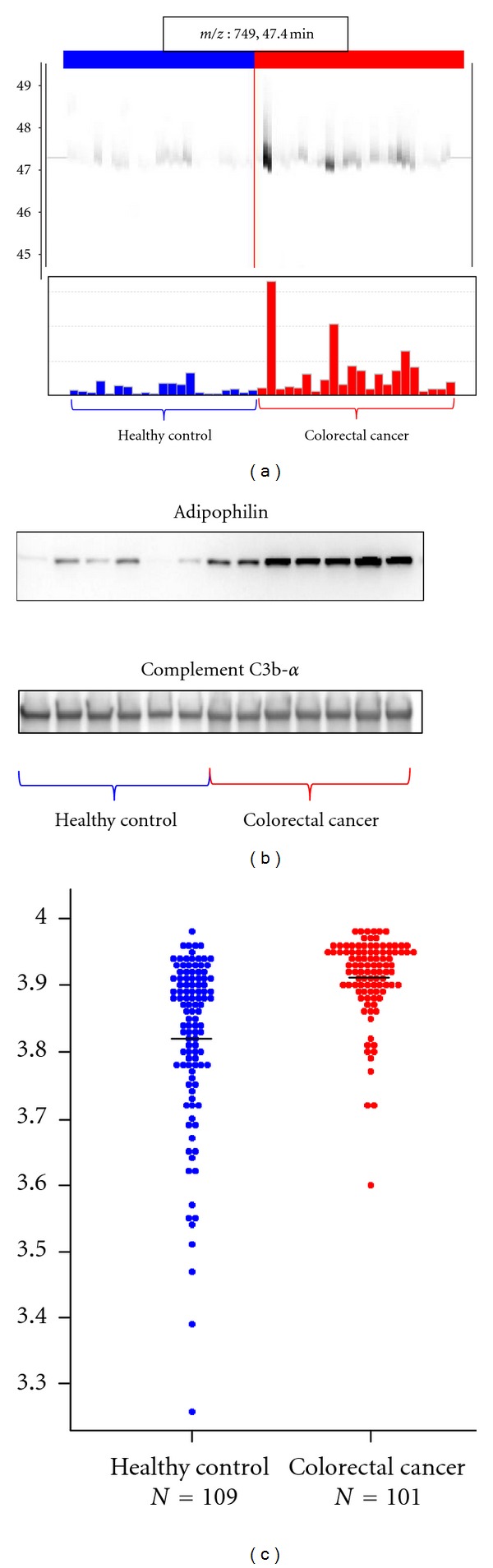
Discovery and validation of adipophilin as a colorectal cancer biomarker (partially changed from [16]). (a) 2DICAL images of the peak (*m/z*, 749; RT, 47.4 min) with coordinates RT versus patients (upper) and intensity versus patients (lower). Red indicates samples from colorectal cancer patients, and blue indicates samples from healthy controls. (b) Western blot of adipophilin and the loading control Complement C3b-*α*. (c) Large-scale RPPA validation of the plasma level of adipophilin using hundreds of clinical samples.

Pretreated samples were subjected to LC/MS and analyzed by 2DICAL. A total of 53009 peptide peaks were detected, and 103 peaks with an AUC of >0.800 were differentially expressed in healthy controls and colorectal cancer patients. Of these, 6 proteins were annotated by database search of tandem mass spectra. The 749 *m*/*z* (RT 47.4 min) peak represents a fragment of adipophilin specifically present in colorectal cancer patients (0.814 in AUC; Figure 4(a)).

**Biomarker Validation**

Small-scale confirmation of adipophilin identification and differential expression was done by immunoblotting using an anti-adipophilin antibody (Figure 4(b)). For large-scale validation, 323 plasma samples were randomly plotted into ProteoChip glass slides for RPPA and blotted with an anti-adipophilin antibody. Differential expression of adipophilin between plasma samples from colorectal cancer patients and from healthy controls was significant (*P* = 5.49 × 10^−10^, Welch *t*-test; Figure 4(c)).

### 4.3. Biomarker for Adverse Effects in Pancreatic Cancer following Chemotherapy

#### 4.3.1. Haptoglobin [17]

**Objective**

Prediction for the adverse effect of pancreatic cancer chemotherapy.

**Samples**

A total of 47 plasma samples collected from patients with pancreatic ductal adenocarcinomas and treated with gemcitabine (2′,2′-difluorodeoxycytidine) monotherapy (25 with severe adverse effects (AEs) and 22 without) were used for biomarker discovery, and 253 plasma samples and 52 serum samples were collected from patients with pancreatic ductal adenocarcinomas treated by gemcitabine monotherapy for validation.

**Sample Preparation**

Samples were treated with a 12 abundant plasma protein removal column to reduce plasma protein complexity.

**Biomarker Discovery**

**Figure 5:**
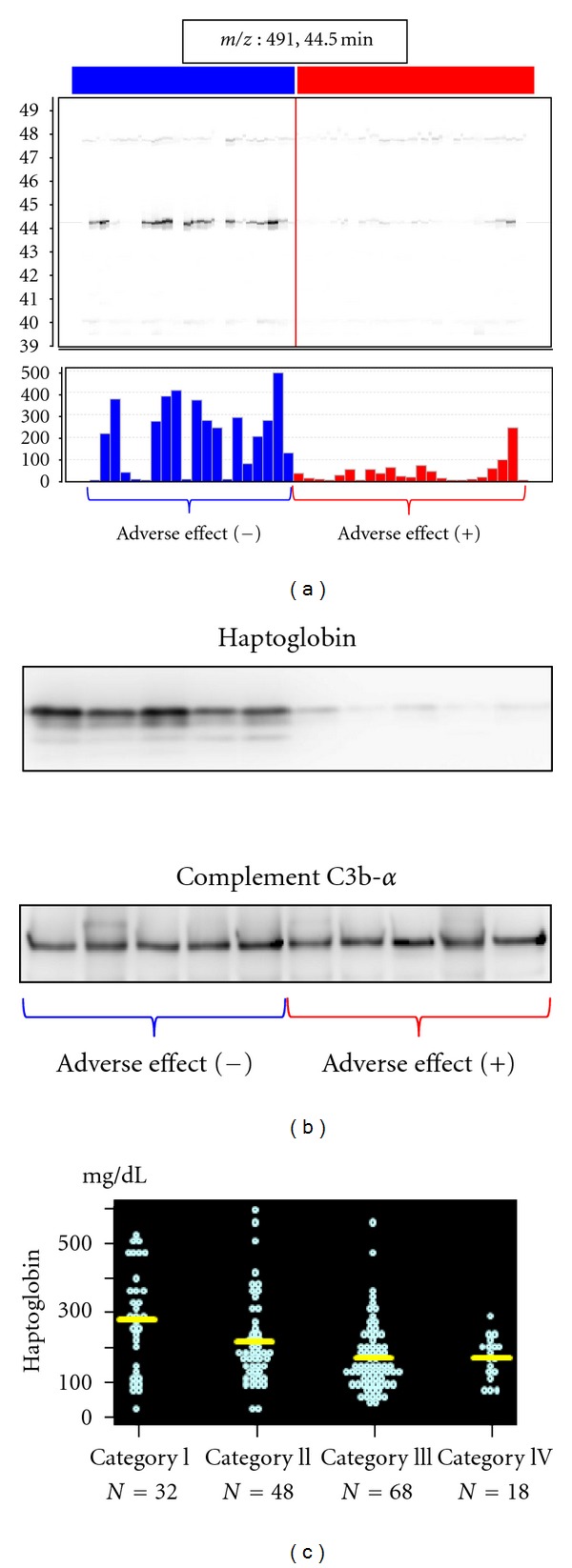
Discovery and validation of haptoglobin as a biomarker for adverse effects in pancreatic cancer following chemotherapy (partially changed from [17]). (a) 2DICAL images of the peak (*m*/*z*, 409; RT, 44.5 min) with coordinates RT versus patients (upper) and intensity versus patients (lower). Red indicates samples from pancreatic cancer patients with severe AEs following chemotherapy, and blue indicates samples from pancreatic cancer patients without AEs following chemotherapy. (b) Western blot of haptoglobin and the loading control Complement C3b-*α*. (c) Large-scale immunonephelometric validation of the plasma level of haptoglobin using hundreds of clinical samples. The adverse effects were categorized in four grades according to the degree of neutropenia and thrombocytopenia. Haptoglobin concentration decreased according to the increase of the adverse effect severity.

Samples were subjected to LC/MS and analyzed by 2DICAL. A total of 60,888 peaks were detected and 757 peaks differed significantly between patients with severe AEs and patients without AEs (*P* < 0.001, Welch *t-*test). Among these, the peak with highest value to discriminate patients with severe AEs from those without AEs was annotated as haptoglobin. The haptoglobin fragment peak of 491 *m*/*z* (RT 44.5 min) is shown in Figure 5(a).

**Biomarker Validation**

Small-scale confirmation of haptoglobin identification and differential expression was confirmed by immunoblotting using an anti-haptoglobin antibody (Figure 5(b)). Haptoglobin concentration in 305 plasma and serum samples was measured by immunonephelometry. The severity of AE severity inversely correlated with the concentration of haptoglobin (Figure 5(c)).

### 4.4. Biomarker for Predicting Survival of Pancreatic Cancer Patients following Chemotherapy

#### 4.4.1. *α*1-Antitrypsin [11]

**Objective**

Prediction of the survival for pancreatic cancer chemotherapy.

**Samples**

A total of 60 plasma samples collected from patients with pancreatic ductal adenocarcinomas and treated by gemcitabine monotherapy (29 with short-term survival and 31 with long-term survival) were used for biomarker discovery, and 304 samples collected from patients with pancreatic ductal adenocarcinomas and treated by gemcitabine monotherapy were used for validation.

**Sample Preparation**

Samples were treated with 12-abundant-plasma-protein removal column to reduce plasma protein complexity.

**Biomarker Discovery**

**Figure 6:**
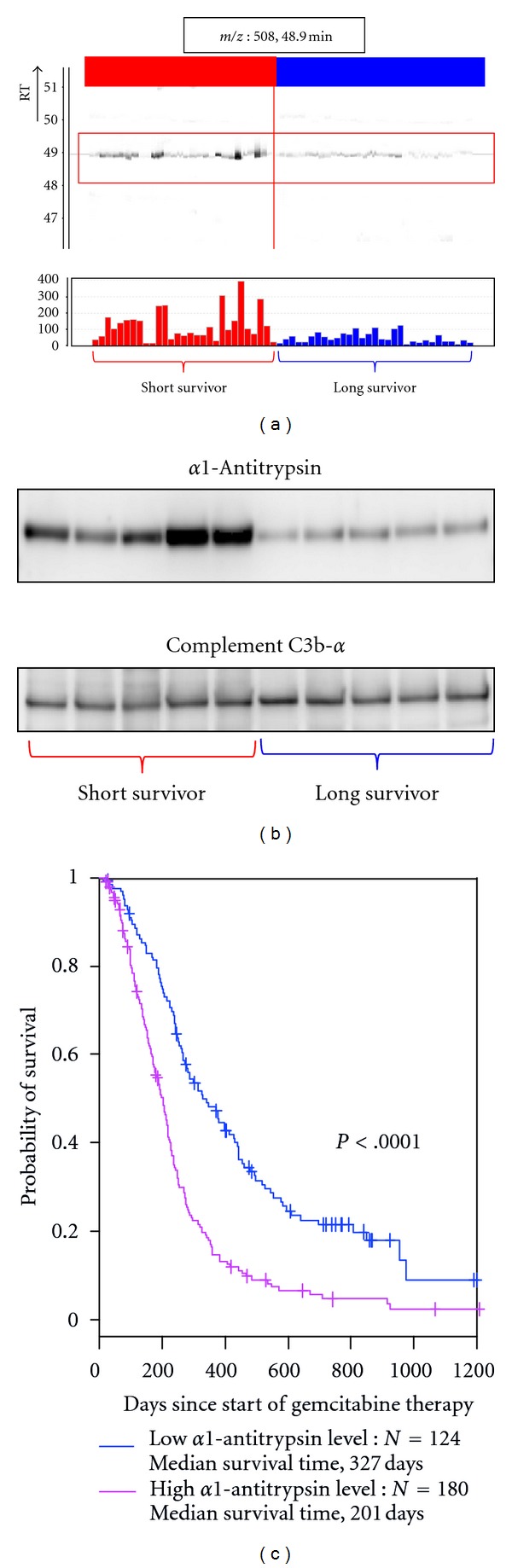
Discovery and validation of *α*1-antitrypsin as a biomarker for predicting survival of pancreatic cancer patients following chemotherapy (partially changed from [11]). (a) 2DICAL images of the peak (*m*/*z*, 508; RT, 48.9 min) with coordinates RT versus patients (upper) and intensity versus patients (lower). Red indicates samples from pancreatic cancer patients with short-term survival, and blue indicates samples from pancreatic cancer patients with long-term survival. (b) Western blot of *α*1-antitrypsin and the loading control Complement C3b-*α*. (c) Large-scale RPPA validation of the plasma level of *α*1-antitrypsin using hundreds of clinical samples. Survival curve was significantly better in the group of low *α*1-antitrypsin level than that of high *α*1-antitrypsin level.

Samples were subjected to LC/MS and analyzed by 2DICAL. A total of 45227 peaks were detected, and 637 peaks differed significantly between patients with long-term survival and those with short-term survival (*P* < 0.001, Welch *t-*test). The peptide peak that best discriminated patients with short-term survival from those with long-term survival (*P* = 2.57 × 10^−4^) at 491 *m/z* (RT 44.5 min) was annotated as a fragment of a1-antitrypsin (Figure 6(a)).

**Biomarker Validation**

Small-scale confirmation of *α*1-antitrypsin identification and differential expression was done by immunoblotting using an anti-*α*1-antitrypsinantibody (Figure 6(b)). For large-scale validation, 304 samples were randomly plotted into ProteoChip glass slides for RPPA and blotted with antibody to *α*1-antitrypsin. Improved survival of patients with pancreatic ductal adenocarcinoma treated by gemcitabine monotherapy correlated with low blood concentrations of *α*1-antitrypsin (Figure 6(c)).

## 5. Conclusions

We have established a comprehensive method for identifying blood biomarkers, which covers all aspects of analysis from sample recruitment to biomarker discovery and validation. The next stage in the development of these novel biomarkers is to test them in a clinical context. The proteomics approach for blood biomarker discovery identifies a new function for common proteins such as these biomarkers. With technological advances in sample preparations, resolution and sensitivity of mass spectrometer, and methods for the identification of proteins from mass spectra, we can expect to discover biomarkers existing in much smaller amount or those with new structures in the future. We also expect that large-scale validation of biomarkers discovered using mass spectrometer will be conducted by MRM/SRM. 2DICAL is applicable not only for proteomics but also for metabolomics or glycomics and has a great potential for identifying disease-associated post-translational protein modifications. 2DICAL will evolve along with technological advances and contribute the discovery of new biomarkers in future.

## Supplementary Material

The value of sensitivity and specificity, the receiver operator characteristic (ROC) curves and areas under the curves (AUC) for each biomarker. The optimal cut-off point was chosen using Youden's Index.
