# Supplementary material for: Biomarker Discovery of Pancreatic and Gastrointestinal Cancer by 2DICAL: 2-Dimensional Image-Converted Analysis of Liquid Chromatography and Mass Spectrometry
Source: Int J Proteomics. 2012 Jul 10;2012:897412. doi: 10.1155/2012/897412 (PMC3400370; doi:10.1155/2012/897412)

Prolyl hydroxylated α-fibrinogen

Sensitivity :0.706
Specificity:0.726

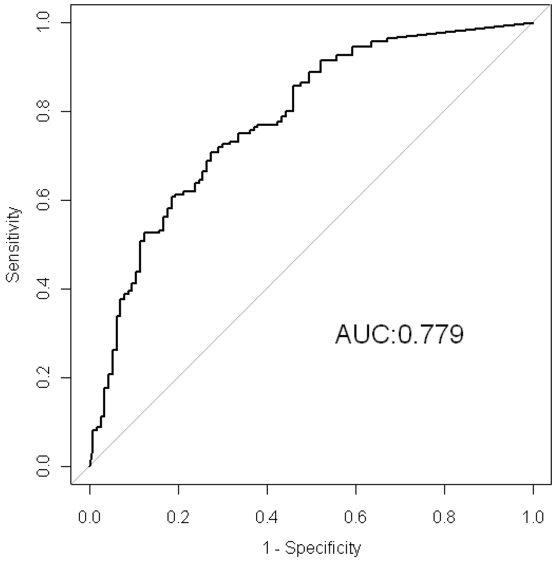

CXC Chemokine Ligand 7

Sensitivity :0.764
Specificity:0.793

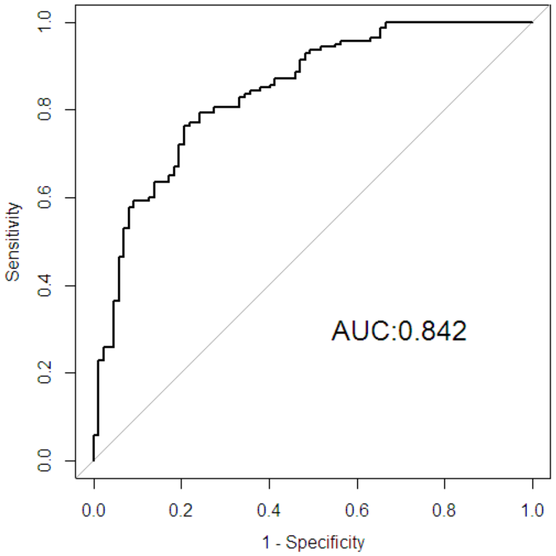

Complement Component 9

Sensitivity:0.670
Specificity:0.691

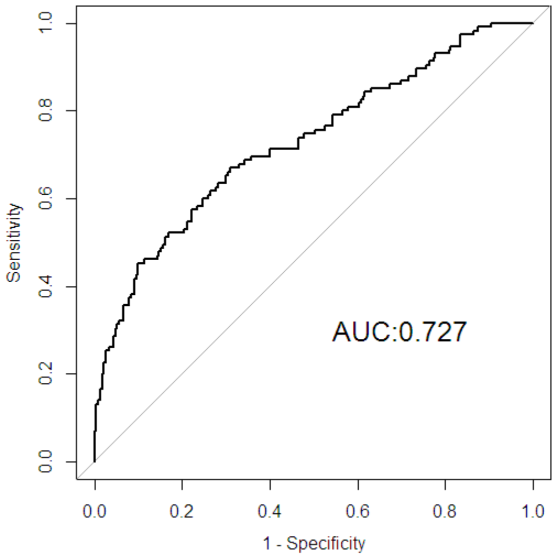

Adipophilin

Sensitivity:0.680
Specificity:0.862

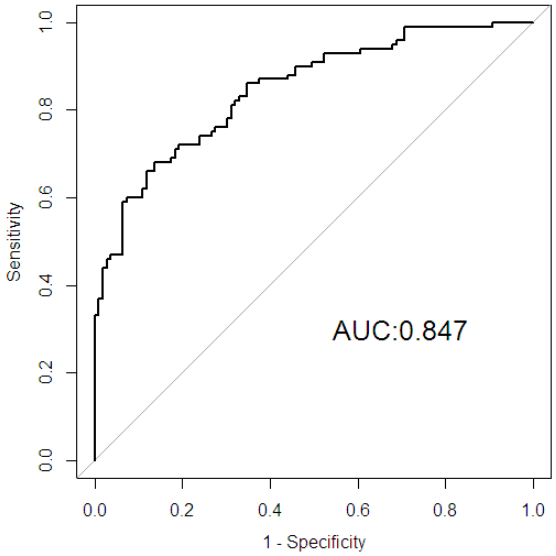

Supplement: Supplementary file 1 — The value of sensitivity and specificity, the receiver operator characteristic (ROC) curves and areas under the curves (AUC) for each biomarker. The optimal cut-off point was chosen using Youden's Index. [file 897412.f1.pdf]
